# Extracellular Dopamine Levels in Nucleus Accumbens after Chronic Stress in Rats with Persistently High vs. Low 50-kHz Ultrasonic Vocalization Response

**DOI:** 10.3390/brainsci11040470

**Published:** 2021-04-08

**Authors:** Kadri Kõiv, Kai Tiitsaar, Karita Laugus, Jaanus Harro

**Affiliations:** 1 Institute of Psychology, University of Tartu, Ravila 14A, 50411 Tartu, Estonia; Kadri.Koiv@ut.ee (K.K.); Kai.Tiitsaar@ut.ee (K.T.); 2 Chair of Neuropsychopharmacology, Institute of Chemistry, University of Tartu, Ravila 14A, 50411 Tartu, Estonia; Karita.Laugus@ut.ee

**Keywords:** 50-khz ultrasonic vocalizations (USVs), individual differences, chronic variable stress (CVS), dopamine, nucleus accumbens, microdialysis

## Abstract

Fifty-kHz ultrasonic vocalizations (USVs) in response to an imitation of rough-and-tumble play (‘tickling’) have been associated with positive affective states and rewarding experience in the rat. This USV response can be used as a measure of inter-individual differences in positive affect. We have previously shown that rats with persistently low positive affectivity are more vulnerable to the effects of chronic variable stress (CVS). To examine whether these differential responses are associated with dopaminergic neurotransmission in the nucleus accumbens (NAc), juvenile male Wistar rats were categorized as of high or low positive affectivity (HC and LC, respectively), and after reaching adulthood, extracellular dopamine (DA) levels in the NAc shell were measured using in vivo microdialysis after three weeks of CVS. Baseline levels of DA were compared as well as the response to K^+^-induced depolarization and the effect of glial glutamate transporter EAAT2 inhibition by 4 mM l-trans-pyrrolidine-2,4-dicarboxylate (PDC). DA baseline levels were higher in control LC-rats, and stress significantly lowered the DA content in LC-rats. An interaction of stress and affectivity appeared in response to depolarization where stress increased the DA output in HC-rats whereas it decreased it in LC-rats. These results show that NAc-shell DA is differentially regulated in response to stress in animals with high and low positive affect.

## 1. Introduction

Rats emit and perceive ultrasonic vocalizations (USVs) to communicate emotional states and manage social contacts with conspecifics [1,2,3]. The 50-kHz type of USVs occur during various appetitive situations such as mating, rough-and-tumble play, and experimenter-administered playful tactile stimulation that mimics the natural rough-and-tumble play, also called ‘tickling’ [4,5]. The production of 50-kHz USVs is strongly related to the activity in the ascending mesolimbic dopaminergic projections from the ventral tegmental area (VTA) to the nucleus accumbens (NAc) [1,6], and it is modulated by several neurochemical systems, e.g., noradrenergic [7], serotonergic [8], opioidergic [9], adenosinergic [10], and glutamatergic [11] receptors.

Animal models for positive emotionality are scarce but understanding the neurobiology of positive affect could make a unique contribution to drug development for affective disorders [12]. We have previously demonstrated that when juvenile rats are individually housed after weaning, daily tickling for two weeks reveals stable inter-individual differences in the level of 50-kHz USVs that persist into adulthood [13]. Male rats with low 50-kHz USV response (LC-rats) are more susceptible to chronic variable stress (CVS) than rats with persistently high 50-kHz USV response (HC-rats) as reflected by changes in behavior, oxidative metabolism in brain regions relevant for emotion processing, higher corticosterone response, and reduced sensitivity to amphetamine treatment [14,15,16].

There is ample evidence that stress modifies the functioning of the mesolimbic dopamine system. Acute stressors such as restraint stress, forced swimming, or intermittent foot-shock increase dopamine (DA) release in the NAc [17,18,19,20] showing that the mesolimbic DA system is activated by stress. Chronic stress regimens have been shown to reduce the basal and to blunt the stimulated overflow of DA in the shell part of NAc [21] and to blunt DA release in response to feeding of a palatable food [22,23]. Also, resilience to stress is associated with dopamine function in the NAc (e.g., [24]). How the role of accumbal DA in stress resilience relates to the positive affect is not known. Interestingly, pharmacological inhibition of corticosterone synthesis reduced 50-kHz USVs elicited either by social contact or amphetamine treatment, even though corticosterone itself did not lead to 50-kHz USV emission [25], but reversed the stress-induced decrease in 50-kHz USVs in another study [26].

Several neural pathways relate to the dopamine reward signals in the NAc, with glutamatergic excitatory innervation possibly exerting the broadest influence. The NAc receives dense glutamatergic innervation from the prefrontal cortex, amygdala, and hippocampus, among many other regions, and distinct glutamatergic inputs may serve different roles in regulating reward and aversion [23,27]. Interaction of dopamine and glutamate in the NAc regulates the shifting between pleasurable activities and responses to stressful environments [28]. Previous studies on the 50-kHz USV production have suggested that glutamate-elicited USVs are dependent on DA [29] and social-encounter-elicited vocalizations cause simultaneous alterations in VTA glutamate and dopamine [30]. It is thus of interest whether glutamate levels contribute to any eventual role of accumbal dopamine in the interaction of stress response with positive affectivity.

In the present study we compared the effect of chronic stress on basal and depolarization-dependent dopamine levels in the NAc shell in rats with higher vs. lower traits of positive affect. Further, as this area also receives glutamatergic innervations, glutamate-mediated modulation of DA levels using a blockade of glutamate transporters by retrodialysis of l-trans-pyrrolidine-2,4-dicarboxylate (PDC) was used.

## 2. Materials and Methods

### 2.1. General Procedure

**Figure 1:**
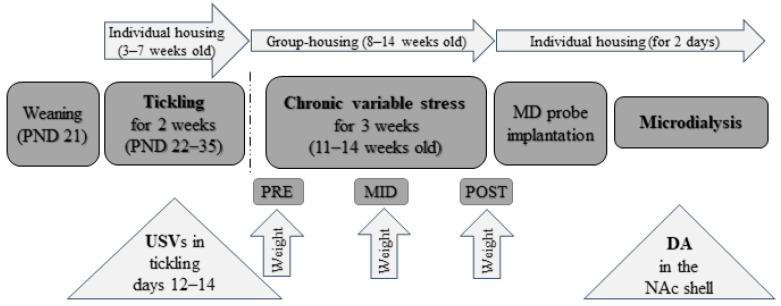
The general timeline of experimental procedures. DA, dopamine; MD, microdialysis; NAc, nucleus accumbens; PND, postnatal day; USVs, ultrasonic vocalizations.

The animals were bred at the local animal house, using parent Wistar rats (Harlan Laboratories, the Netherlands). Rat pups were weaned at 21 days of age (experiment timeline in Figure 1) and single-housed in standard transparent polypropylene cages with wood-chip bedding in a temperature-controlled colony room (20–22 °C) under a 12:12 light/dark cycle, with lights on at 08:00 h. Rats had free access to tap water and food pellets (diet R70, Lactamin AB, Sweden), except during testing. Tickling started the day after single-housing and only male pups were used for the experiment. The rats were group-housed (four per group) three weeks after the end of the tickling sessions, which had lasted for 14 days, and they remained so until the day of surgery. The CVS regimen started when the rats were 11 weeks old. The rats were weighed before stress, 11 days into stress, and after the end of the CVS regimen. One day after the end of the stress period, the microdialysis probe was implanted and the next day, the microdialysis experiment in awake and freely moving animals was carried out. The experimental protocol was approved by the Animal Experimentation Committee at the Estonian Ministry of Agriculture (7.2–11/10).

### 2.2. Tickling

Tickling procedure was carried out on postnatal days 22–35, as previously [13,16]. Animals were individually transported to the adjacent experiment room, transferred from their home cage into a smaller (30 × 15 × 13 cm) plastic cage without bedding and given 15 s for habituation. This was followed by 15 s of tickling, which is a playful manual stimulation resembling natural rough-and-tumble play in juvenile rats, by the experimenter. Separated by 15 s pauses, altogether four 15 s sessions of tactile stimulation were given over 2 min, after which animals were returned to their home cage and the test cage was cleaned with a damp tissue. The tickling session consisted of stimulating the rat with one hand and included vigorous alternating finger movements on the back and scruff, rapidly turning the animal over on the back whilst stimulation was administered on the ventral surface, followed by release after a few seconds [4,5].

During the tickling sessions, an ultrasound microphone (Avisoft Ultra Sound Gate 116–200, Avisoft Bioacoustics, Berlin, Germany) was located about 20 cm from the floor of the tickling cage, recording USVs with a sampling rate of 300 kHz in 16-bit format on a computer hard drive. The files were later analyzed with Avisoft SASLab Pro (Avisoft Bioacoustics, Berlin, Germany) software, creating spectrograms using the Fast Fourier Transform algorithm (1024 FFT length, 75% frame, Hamming window, and 75% time window overlap). Spectral data of the sound recordings were manually cleaned from noise after which automatic scoring was applied to count the number of 50-kHz USVs with frequencies over 38 kHz during the tickling stimulation time (altogether 1 min). The rats were divided into groups with high and low levels of 50-kHz USVs by the median split of the average response on days 12–14 of tickling, providing the HC and LC groups. The HC-rats emitted on average 1.7 times more 50-kHz vocalizations compared to the LC-rats (HC, 268 ± 5; LC, 159 ± 9; F(1,27) = 114.1, *p* < 0.0001)

### 2.3. Chronic Variable Stress Regimen (CVS)

The chronic variable stress (CVS) regimen was applied to half of the animals for 3 weeks, as carried out previously [16]. In brief, the protocol comprised of six short and five long moderately unpleasant environmental and social stressors that were used intermittently, one per day, each one maximally once per week. The control rats lived undisturbed in the colony room. The CVS-regimen was applied by one group-housing cage at a time (four rats) and timed so that the 21-day stress regimen could be followed by microdialysis. The stressors were (in order of occurrence), movement restriction in a small plastic compartment (25 × 9 cm, 2 h), cage tilt at 45° (24 h), placement on a round 10-cm diameter platform elevated 75 cm from ground with strong illumination (900 lx, 30 min), wet bedding (24 h), cold environment (4 °C, 1 h), overcrowding (eight rats in a novel home-cage, 24 h), short immobilization with a thick glove (1 min), stroboscopic light during the predicted dark period (10–50 Hz; 12 h), tail-pinch with a clothespin placed 1 cm distal from the base of tail (5 min), strong illumination (900 lx) during predicted dark phase (12 h), and loud white noise (1 h). These stressors were administered in a separate room during the light phase of the cycle (except for the stressors that lasted overnight), while the control rats stayed undisturbed in the colony room.

### 2.4. Microdialysis Procedure

Microdialysis was carried out essentially as previously described [31]. The animals were anaesthetized with combined solution of ketamine and medetomidine (45 mg/kg and 0.3 mg/kg, IP, respectively) and mounted in a Kopf stereotactic frame while being kept on a heating-pad. A self-made concentric Y-shaped microdialysis probe with 8-mm shaft length and 1.5-mm active tip was implanted at 20° angle aimed at the left NAc shell according to the following coordinates: anterior-posterior (AP) +1.7; medial-lateral (ML) −2.0; dorsal-ventral (DV) −8.0, according to the brain atlas by Paxinos and Watson [32]. The dialysis membrane used was polyacrylonitrile/sodium methallyl sulphonate copolymer (Filtral 12; innerdiameter(ID) 0.22 mm; outerdiameter (OD) 0.31 mm; AN 69, Hospal, Bologna, Italy). Two stainless steel screws and dental cement were used to fix the probe to the scull, with 1% lidocaine being used for wound infiltration anesthesia, and the rats were placed in 21 cm × 36 cm × 18 cm individual cages in which they remained throughout the microdialysis experiment. The rats were left to recover and on the next day, the microdialysis procedure was conducted in awake, freely moving animals. The microdialysis probe was connected to a syringe pump (SP101, World Precision Instruments, Inc., Sarasota, USA) and cooling microfraction collector (4 °C, CMA/142, CMA Microdialysis AB, Solna, Sweden) and perfused with Ringer solution (140 mM NaCl, 4 mM KCl, 1.2 mM CaCl_2_, 1 mM MgCl_2_, 1 mM Na_2_HPO_4_, 0.2 mM NaH_2_PO_4_, pH = 7.21) at a constant rate of 1.5 μL/min. Connections to the infusion pump and sample collection loop or microfraction collector were made with flexible FEP tubing (i.d. 0.12 mm, AgnTho’s AB, Lidingö, Sweden). After connecting the animal to the microdialysis system, the perfusate was discarded during the first 60 min to allow stabilization. Twenty microdialysis samples were collected with 20 min intervals.

During microdialysis, two manipulations were used to stimulate the dopamine output in the NAc shell. KCl stimulation imitates neural excitation and can be applied to study the potential to release a neurotransmitter from the neurons in response to stimulation. The concentration of KCl appropriate for depolarization was based on previous work [33,34]. In order to investigate the role of glutamate in dopamine release after stress, we used glutamate re-uptake inhibition by perfusion with 4 mM l-trans-pyrrolidine-2,4-dicarboxylate (PDC), an effective EAAT2 inhibitor [35,36,37].

Four baseline samples were collected, followed by 1-h perfusion with 50 mM KCl solution (samples 5–7). Next, Ringer solution was used for perfusion in samples 8–12, after which 4 mM PDC was infused for an hour (samples 13–15). For the final five samples, the system was switched back to Ringer solution. Then, 22.5 μL of the dialysate buffered in 7.5 μL of 0.02 M acetic acid was used and the samples were kept at −80 °C until measurement. At the end of the microdialysate collection, the rats were decapitated and the brains were removed and kept at −80 °C. The brains were sectioned on a cryostatic microtome (Microm GmbH, Walldorf, Germany) and probe placements were determined according to the atlas of Paxinos and Watson [32]. Only animals with correct probe placements were included in the HPLC analysis (*n* = 33).

### 2.5. Quantification of Dopamine in Microdialysates

The quantity of dopamine in the microdialysis samples was determined by high performance liquid chromatography (HPLC) with electrochemical detection. The chromatography system consisted of a Shimadzu LC-10AD series solvent delivery pump (Shimadzu Corporation, Kyoto, Japan), a Luna C18(2) 5 μm column (150 × 2 mm) (Phenomenex Inc., Torrane, CA, USA) kept at 30 °C and a Decade II digital electrochemical amperometric detector (Antec Scientific, Zoeterwoude, The Netherlands) with electrochemical flow cell VT-03 (2 mm GC WE, ISAAC reference electrode, Antec Scientific, Zoeterwoude, The Netherlands). The mobile phase consisted of 0.05 M sodium citrate buffered to pH 5.3, 2 mM KCl, 0.02 mM EDTA, 3.5 mM sodium octyl sulfonate, and 14% acetonitrile. The mobile phase was filtered through a 0.22-µm pore size filter (MilliporeSigma, Burlington, MA, USA) and was pumped through the column at a rate of 0.2 mL/min. DA eluted from the column was measured with a glassy carbon working electrode maintained at a potential of +0.4 V versus Ag/AgCl reference electrode. Dopamine content (fmol/22.5 μL) was calculated using external standard solutions (Sigma, Buchs, Switzerland).

### 2.6. Data Analysis

Microdialysis data were analyzed with repeated measures (time) ANOVA with affectivity (HC/LC) and stress (stress vs. control) as independent variables. Baseline dopamine levels in the NAc shell were calculated for every individual animal as the average (mean ± standard error of the mean, SEM) of the samples 2–4. (The first baseline sample was excluded owing to the large variability, hence making the effective stabilization period 80 min.) In order to estimate the magnitude of stimulation with KCl and PDC, the area under the curve (AUC) for samples 5–11 and 13–20 was calculated for every individual animal, respectively, using OriginPro 9 (OriginLab Corporation, Northampton, MA, USA). Body weight data, data about the baseline levels of accumbal dopamine, and AUC data were analyzed with two-factor ANOVA with affectivity (HC vs. LC) and stress (stress vs. control) as independent variables. Where appropriate, Fisher’s Protected Least Significant Difference (PLSD) test was used as a post hoc test. Statistical analysis was performed using StatView 5.0. (SAS Institute Inc., Cary, NC, USA) and Statistica 8.0. (StatSoft Inc., Tulsa, OK, USA). The data from four animals were left out of the analysis as their DA levels differed from their group averages by more than three standard deviations.

## 3. Results

### 3.1. Stress Effect on Body Weight Gain

**Figure 2:**
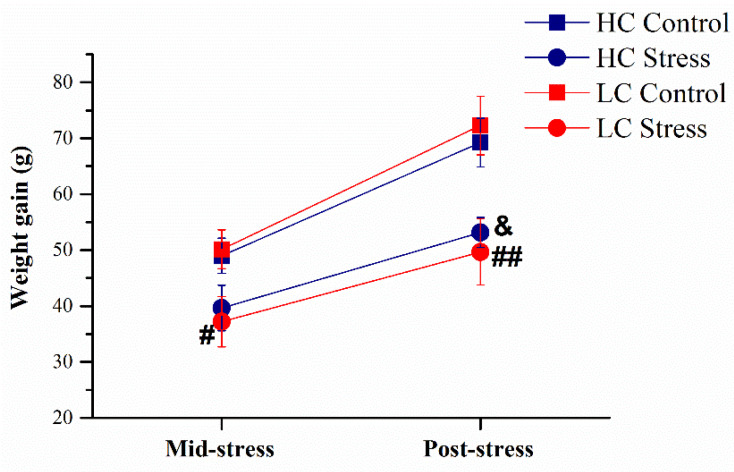
Cumulative weight gain in the middle (day 11 of stress) and after the three-week stress regimen (average ± SEM). #, ## *p* < 0.05, 0.01 vs. LC-control; and *p* < 0.05 vs. HC-control. HC-controls: *n* = 8; HC-stress, *n* = 8; LC-controls, *n* = 7; and LC-stress, *n* = 6. HC, high 50-kHz USV rats, LC, low 50-kHz USV rats.

On average, the three-week-long stress regimen decelerated weight gain of comparable magnitude in HC- and LC-rats, by 23% in HC- and by 31% in LC-rats (main effect of stress F(1,25) = 18.23, *p* < 0.001, Figure 2).

### 3.2. Dopamine Content in Microdialysates

**Figure 3:**
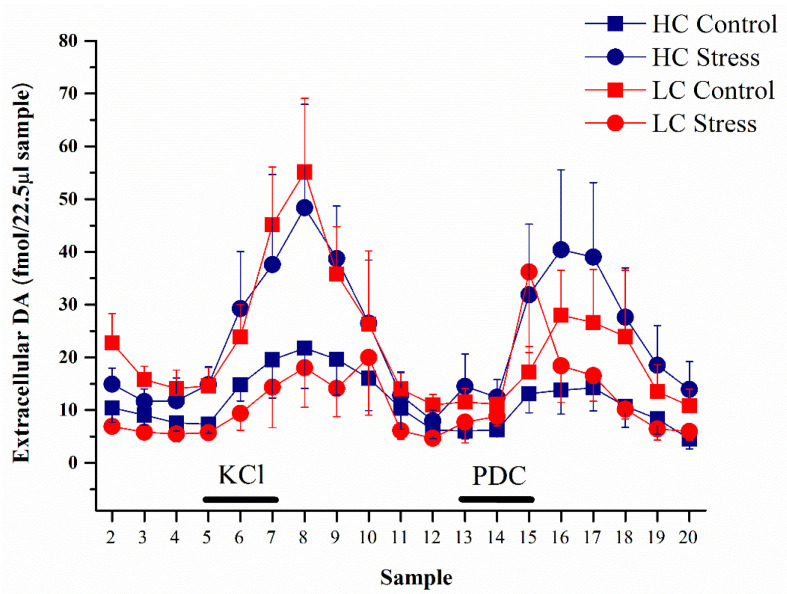
Extracellular dopamine levels in the NAc shell as measured during microdialysis (average + or-SEM). Baseline (samples 2–4), perfusion with 50 mM KCl solution (samples 5–7), and perfusion with 4 mM l-trans-pyrrolidine-2,4-dicarboxylate (PDC) (samples 13–15). HC-controls, *n* = 8; HC-stress, *n* = 8; LC-controls, *n* = 7; and LC-stress, *n* = 6. HC, high 50-kHz USV rats and LC, low 50-kHz USV rats.

Dopamine levels in microdialysate samples differed group-wise across the experiment and the effect of stress on DA levels was dependent on the affectivity phenotype (repeated measures ANOVA main effect of time (F(18,450) = 8.0, *p* < 0.001), interaction of affectivity × stress (F(1,25) = 5.8, *p* < 0.05), and interaction of affectivity × stress × time (F(18,450) = 1.8, *p* < 0.05), Figure 3).

**Figure 4:**
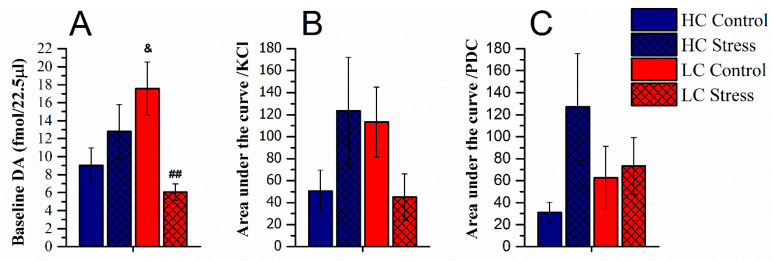
Extracellular dopamine levels in the NAc shell as measured during microdialysis (average ± SEM). (**A**) Average in baseline dopamine content (samples 2–4), (**B**) area under the curve (AUC) during and after perfusion with 50 mM KCl solution (samples 5–11), (**C**) area under the curve (AUC) during and after perfusion with 4 mM PDC (samples 13–20). ## *p* < 0.01 vs. LC-control; and *p* < 0.05 vs. HC-control. HC-controls, *n* = 8; HC-stress, *n* = 8; LC-controls, *n* = 7; and LC-stress: *n* = 6. HC, high 50-kHz USV rats and LC, low 50-kHz USV rats.

In detail, the baseline extracellular levels of dopamine (average in samples 2–4) in the NAc shell were as follows: HC-controls, 9.0 ± 2.0 fmol/22.5 μL sample (*n* = 8) and HC-stress, 12.8 ±3.0 fmol/22.5 μL sample (*n* = 8); LC-controls, 17.6 ± 3.0 fmol/22.5 μL sample (*n* = 7) and LC-stress, 6.1 ± 0.9 fmol/22.5 μL sample (*n* = 6). LC-controls had higher baseline levels compared to HC-rats, and stress reduced baseline DA levels only in the LC-rats while rather tending to increase dopamine levels in the HC-rats (interaction of affectivity × stress F(1,25) = 9.3; *p* < 0.01, Figure 4A).

There was an increase in DA levels in response both to KCl and PDC (Figure 4B,C). To compare the effect of KCl on dopamine release, for every rat the area under the curve was calculated for samples 5–11. Again, affectivity and stress interaction on dopamine output was revealed, as stress heightened the DA levels for HC whereas it lowered it in LC-animals (F(1,25) = 4.2; *p* < 0.05, Figure 4B). However, post hoc tests failed to show significant differences between individual groups, because while the difference between groups was almost perfectly reversed by stress, the within-group variability was substantial. After PDC retrodialysis (samples 13–20), a tendency for higher output of DA in stressed HC-rats appeared, but this effect failed to reach statistical significance (NS; Figure 4C).

## 4. Discussion

The results show that the positive affectivity trait is associated with dopamine levels in the NAc, and that chronic stress can affect the dopamine output in the NAc shell differently depending on the positive affectivity phenotype as measured by tickling-induced 50-kHz vocalizations.

Stress prevented weight gain significantly in both HC- and LC-rats, showing the effectiveness of the chronic stress regimen used in this study. In our previous experiments stressed LC-rats gained weight significantly less as compared to the HC-rats [14,15]. In the present experiment the reduction of weight gain by stress in the LC-rats and HC-rats was not significantly different, while a tendency to lower weight gain in comparison with the corresponding control group was observed at mid-stress. In the present experiment weight gain was somewhat lower in all groups, possibly leading to reduced capacity to discriminate between LC- and HC-rats at this relatively mild stress regimen.

Overall, without stress, extracellular levels of DA were higher in LC-rats than in HC-rats. Individual differences in DA release in the NAc shell have been reported by many studies with variable design [38,39]. The mechanisms that have been found to underlie such differences include the size of the presynaptic pools of DA, the tyrosine hydroxylase activity, and the concentration of vesicular monoamine transporters, as well as the level of function of the noradrenergic neurotransmission [40]. It will need to be established in further experiments which mechanism(s) could underlie the higher extracellular DA levels in LC-rats. Microinjection of DA into the NAc shell elicited 50-kHz USVs [41]. This study, however, also reported that direct application of DA to the accumbens was less efficacious in evoking 50-kHz USVs than amphetamine if DA uptake was not additionally inhibited. Speculatively, LC-rats may have constitutively lower DA reuptake, causing functional desensitization of postsynaptic response to DA by constitutively higher extracellular levels of dopamine. At least three subtypes of DA receptors, D_1_, D_2_, and D_3_, are involved in the emission of 50-kHz USVs [42]. Interestingly, administration of amphetamine into the NAc shell did elicit 50-kHz USVs in the Long Evans rat line with high vocalization rate in response to social stimulation, but not in the corresponding line with low response [43]. This may suggest that in LC-rats, the 50-kHz USV response can be uncoupled of DA-ergic neurotransmission in the NAc.

Chronic stress reduced extracellular DA levels in LC-rats. In our previous studies, chronic variable stress produced, in male LC-rats 22-kHz USVs, lower sucrose consumption, higher increase in immobility in the forced swimming test, and increase of oxidative metabolism in several brain areas (but not the accumbens) [13], higher levels of corticosterone in an acute stressful condition, robustly higher extracellular levels of 5-HT in the hippocampus if 5-HT reuptake was blocked [15], and larger reduction of amphetamine stimulation of 50-kHz USVs and locomotion [16]. Altogether these findings support the notion that low positive affectivity presents a vulnerability to chronic stress. Other investigators using different chronic stress paradigms have also observed reduction of extracellular DA in the NAc shell [21,22,23], so this mechanism appears to serve as a common ground for vulnerability to stress. However, the fact that in HC-rats the stress effect on extracellular DA strongly tended to be exactly the opposite requires an explanation. It may be relevant to consider that in the present experiment all rats had been tickled in early age. Several behavioral differences can be found in both LC- and HC-rats in comparison with not-tickled control rats [13]. In general, tickled rats express less fear and anxiety in adulthood. The HC-rats have particularly reduced anxiety as revealed in the elevated plus-maze test. Thus, a potential for developing higher extracellular DA levels in the NAc in stressful conditions must be acknowledged in the context of combination of low anxiety and high positive affectivity. In one study rats were classified on the basis of behavioral activity in the Porsolt’s forced swimming test and the passive animals were found to produce more 50-kHz USVs in a social encounter [44]; in these rats chronic stress reduced in the NAc shell the levels of the cocaine- and amphetamine-regulated transcript peptide that is known to reduce DA release in this region [45]. While this experiment has substantial differences it appears to associate higher 50-kHz production with the potential of increased DA release after chronic stress.

Amongst the possible mediators of the inverse relationship of extracellular dopamine with stress in LC- vs. HC-rats, the role of the serotonergic system should be considered. Serotonin potentiates the cocaine-enhanced extracellular DA levels in the NAc shell [46] and chronic stress can interfere with the action of serotonin on DA release in the mesoaccumbens pathway [47]. In mice, serotonin controls DA release through 5-HT_2C_ receptors in the NAc core [48], and in the shell, alcohol exposure increased basal extracellular levels of both DA and serotonin, and 5-HT_2C_ receptor blockade reduced alcohol intake [49]. In our previous study, ex vivo measurements in the NAc revealed that while chronic variable stress tended to increase 5-HT turnover in HC-rats, it was the opposite in the case of LC-rats [16]. Serotonin turnover was reduced in the NAc shell by fluoxetine if rats had previously been treated with corticotropin-releasing factor, a major stress signal [50]. Thus, the high behavioral sensitivity to stress of the LC-rats may in part relate to differences in serotonergic function.

Perfusion of PDC increased DA levels in the NAc shell as has been shown before [51,52]. Projections from the VTA to the NAc shell release both DA and glutamate, and glutamatergic inputs are received by the medium spiny neurons of the NAc shell from several other brain regions [53]. Owing to large variability in the individual levels no group-wise difference was statistically significant. It seems, however, worth noting that LC-rats had twice the level of DA increase by inhibition of glutamate uptake as compared to unstressed HC-rats, and no stress effect was observed in LC-rats while the average increase in stressed HC-rats was several-fold as compared to the respective control. A hypothesis that animals with high positive affect will develop a reduction of glutamatergic input to accumbens as an adaptation to stress should be tested.

Limitations of this study include the small number of animals per group, owing to the complex study design. For this reason liberal post-hoc comparisons were applied after statistically significant interaction effects had been revealed. The period of individual housing applied in the study design may raise questions, as single housing has been shown to be stressful to young animals, especially females, depending on the duration of the isolation period [54,55]. In the present experiment, single-housing was employed as it has been shown to substantially increase the ultrasonic vocalization levels in juveniles in response to tickling [4]. During two of the single-housing weeks, a daily session of imitated rough-and-tumble play was applied and this should lessen the effects of stress. Resocialization was applied as soon as the HC/LC groups were assigned based on their 50-kHz vocalization response. A further limitation is the use of only male rats. We have previously studied both female and male rat response to tickling and shown that the separation to HC- and LC-rats applies to both [13]; however, female HC-rats are more prone to elicit 22-kHz ultrasonic vocalizations and their response to chronic stress significantly differs from males [14]. Future studies should address the USV response after stress and dopamine levels in the NAc in females.

A further consideration is the contrast between the stress responses observed in two different behavioral traits relevant to reward. Thus, we have consistently observed that sensitivity to chronic stress is higher in rats with low positive affect as measured by the expression level of 50-kHz USVs [14,15,16]. In the same general laboratory setting we have also consistently found rats that consume less sucrose to be less sensitive to chronic stress [56,57]. This trait-wise dissociation of stress resilience remains to be replicated in animals characterized for both phenotypes, and the underlying mechanisms identified.
